# Endolymphatic space is age-dependent

**DOI:** 10.1007/s00415-022-11400-8

**Published:** 2022-10-05

**Authors:** Marianne Dieterich, Tatjana Hergenroeder, Rainer Boegle, Johannes Gerb, Emilie Kierig, Sophia Stöcklein, Valerie Kirsch

**Affiliations:** 1 Department of Neurology, University Hospital, Ludwig-Maximilians-Universität, Munich, Germany; 2 German Center for Vertigo and Balance Disorders-IFB, University Hospital, Ludwig-Maximilians-Universität, Munich, Germany; 3 Graduate School of Systemic Neuroscience (GSN), Ludwig-Maximilians-Universität, Munich, Germany; 4 Munich Cluster for Systems Neurology (SyNergy), Munich, Germany; 5 Department of Radiology, University Hospital, Ludwig-Maximilians-Universität, Munich, Germany

**Keywords:** Endolymphatic hydrops, Inner ear, Healthy controls, Asymmetry, Endolymphatic space, MRI

## Abstract

Knowledge of the physiological endolymphatic space (ELS) is necessary to estimate endolymphatic hydrops (ELH) in patients with vestibulocochlear syndromes. Therefore, the current study investigated age-dependent changes in the ELS of participants with normal vestibulocochlear testing. Sixty-four ears of 32 participants with normal vestibulocochlear testing aged between 21 and 75 years (45.8 ± 17.2 years, 20 females, 30 right-handed, two left-handed) were examined by intravenous delayed gadolinium-enhanced magnetic resonance imaging of the inner ear (*i*MRI). Clinical diagnostics included neuro-otological assessment, video-oculography during caloric stimulation, and head-impulse test. *i*MRI data analysis provided semi-quantitative visual grading and automatic algorithmic quantitative segmentation of ELS volume (3D, mm^3^) using a deep learning-based segmentation of the inner ear’s total fluid space (TFS) and volumetric local thresholding, as described earlier. As a result, following a 4-point ordinal scale, a mild ELH (grade 1) was found in 21/64 (32.8%) ears uni- or bilaterally in either cochlear, vestibulum, or both. Age and ELS were found to be positively correlated for the inner ear (*r*(64) = 0.33, *p* < 0.01), and vestibulum (*r*(64) = 0.25, *p* < 0.05). For the cochlea, the values correlated positively without reaching significance (*r*(64) = 0.21). In conclusion, age-dependent increases of the ELS should be considered when evaluating potential ELH in single subjects and statistical group comparisons.

## Introduction

Delayed gadolinium (Gd) enhanced magnetic resonance imaging of the inner ear (*i*MRI) enables in vivo verification of endolymphatic hydrops (ELH, [1, 2]). Before its technical development, ELH was thought pathognomonic to Menière’s disease (MD). However, up to now, MD diagnosis is based on clinical diagnostic criteria [3] and only definitely verifiable in post-mortem human temporal bone (*h*TB) histopathologic inquiry [4].

Fittingly, varying degrees of ELH were nearly universally present in subjects with a history of MD, with 97–100% in post-mortem human temporal bones studies [5, 6] and 80% in *i*MRI studies [7–10]. ELH was further consistently detected in other pathologies presenting with episodic vertigo, such as in 36–60% in intralabyrinthine schwannoma [11–14], 60% in bilateral vestibulopathy [15], 59% in otosclerosis [16], 8–30% in vestibular migraine [8, 17, 18], and also in idiopathic intracranial hypertension [19], spontaneous intracranial hypotension [20, 21], or cerebrospinal venous insufficiency [22]. Consequently, ELH's specificity to MD is being revalued, while its pathophysiological relevance within the pathologies mentioned remains unclear to varying degrees.

It is necessary to be aware of asymptomatic ELH prevalence in healthy subjects to assess the relevance of any ELH found. Moreover, possible age-dependent physiological ELS changes should be considered on a single case or group scale. Unfortunately, however, studies on the ELH prevalence within the healthy population have been carried out noticeably less. Due to the more or less invasive nature of the methods able to verify ELH so far, studies on the healthy population to date were frequently replaced by patient groups that were thought not to be associated with ELH. At the same time, studies accompanied by comprehensive vestibulocochlear functional testing are scarce (for an overview cp. Tables 3 and 4). *h*TB studies often lack information on age and concurrent reference of vestibulocochlear function, while most *i*MRI studies are satisfied with the participants' statement that they did not perceive any vestibulocochlear malfunction. The comparison of the results of these studies is further complicated using different ELS quantification methods in *i*MRI [7] and different fixatives or post-mortem hours in *h*TB [23].

The current study investigated age-dependent changes in the ELS in participants with normal vestibulocochlear function (^*vc*^HP) and discussed them given previous studies conducted on ELS in “healthy” participants.

## Materials and methods

### Setting and institutional review board approval

All data were acquired at the interdisciplinary German Center for Vertigo and Balance Disorders (DSGZ) and the Munich University Hospital Neurology Department (LMU), Germany, between 2018 and 2020. Institutional Review Board approval was obtained before the initiation of the study (no. 641–15). Furthermore, all participants provided informed oral and written consent in accordance with the Declaration of Helsinki before inclusion in the study.

### Study population

Thirty-two consecutive inpatients (64 ears; 20 females; aged 21–75 years, mean age 45.8 ± 17.2 years; 31 right-handed, two left-handed) of the Neurology Department without symptoms or underlying pathologies of the peripheral and central vestibulocochlear system underwent MRI with intravenous contrast agent as part of their diagnostic workup and agreed to undergo additional *i*MRI sequences after 4 h. Ethical considerations did not allow us to include healthy volunteers without a medical indication for an iMRI with contrast agent (see limitations for more information). The reasons for the participant’s admission to the clinic included polyneuropathy (*n* = 5), movement disorders (*n* = 5), single small cortical metastases (*n* = 3), epilepsy (*n* = 3), optic nerve neuritis (*n* = 3), spinal inflammatory lesion (*n* = 3), tension headache (*n* = 3), viral meningitis (*n* = 2), subdural hematoma (*n* = 2), idiopathic facial nerve palsy (*n* = 2), and decompensated esophoria (*n* = 1). Cranial MRI findings were age-appropriate or did not interfere with the vestibulocochlear system. The laterality quotient for right-handedness was assessed with the 10-item inventory of the Edinburgh test [24]. The inclusion criterion was age between 18 and 85 years and normal audiovestibular testing to confirm the soundness of their peripheral end-organs and the central vestibular system (see Sect. 2.3). The exclusion criteria were current cochlear or vestibular disorders, a positive history of vertigo, balance, or hearing disorders, any MR- or contrast agent-related contraindications [25], poor image quality, or missing MR sequences.

### Measurement of the auditory, semicircular canal and otolith functions

Participants without vestibulocochlear symptoms (^*vc*^HP) underwent vestibulocochlear testing to confirm the soundness of their peripheral inner ear end-organs. Diagnostic workup included a thorough neurological examination (e.g., history-taking, clinical examination), neuro-orthoptic assessment [e.g., Frenzel glasses, fundus photography, and adjustments of the subjective visual vertical (SVV) for acute vestibular graviceptive dysfunction], video-oculography (VOG) during caloric and head impulse testing (HIT), and pure tone audiometry (PTA).

A tilt of the SVV is a sensitive sign of an acute graviceptive vestibular tone imbalance. SVV was assessed with the subject sitting upright in front of a half-spherical dome with the head fixed on a chin rest [26]. A mean deviation of > 2.5° from the true vertical was considered a pathological tilt of SVV [26].

The impairment of the vestibulo-ocular reflex (VOR) in higher frequencies was measured by HIT [24] using high-frame-rate VOG with EyeSeeCam ([27], EyeSeeTech, Munich, Germany). A median gain during head impulses < 0.8 (eye velocity in °/s divided by head velocity in °/s) was considered a pathological VOR [28].

Furthermore, semicircular canal responsiveness in lower frequencies was assessed by caloric stimulation (CS) with VOG, which was performed for both ears with 30° cold and 44° warm water. Vestibular paresis was defined as > 25% asymmetry between the right- and left-sided responses [29] or the sum of the maximal peak velocities of the slow phase caloric-induced nystagmus for stimulation with warm and cold water on each side < 25°/sec [30]. The caloric asymmetry index (AI_CS_) was calculated based on the slow-phase velocity of the caloric nystagmus ${AI}_{CS }[\%] =\frac{({R}_{33^\circ }+{R}_{44^\circ })-({L}_{33^\circ }+{L}_{44^\circ })}{({R}_{33^\circ }+{R}_{44^\circ })+({L}_{33^\circ }+{L}_{44^\circ })}\times 100.$

Audiological tests consisted of pure-tone audiometry (PTA) by air conduction at 250 Hz to 8 kHz frequencies. PTA was based on both ears’ four-tone average (arithmetic mean) of the thresholds at 0.5, 1, 2, and 3 kHz. Hearing loss was defined as PTA > 25 dB [31]. In all tests, the contralateral ear was masked by adequate noise. All audiometric equipment is regularly recalibrated (every 6 months) according to the local university equipment standard.

### Delayed intravenous gadolinium-enhanced MRI of the inner ear

#### Data acquisition

Participants received a standard dose (0.1 mmol/kg body weight) of gadobutrol (Gadovist^®^, Bayer, Leverkusen, Germany) once and were scanned in a whole-body 3 Tesla MRI scanner (Magnetom Skyra, Siemens Healthcare, Erlangen, Germany) with a 20-channel head coil twice; first directly after intravenous injection, as part of their diagnostic workup, and a second time after 4 h for the *i*MRI sequences. A T2-weighted, 3D-FLAIR (three-dimensional fluid-attenuated inversion recovery) sequence was used to differentiate endolymph from perilymph and bone, and a spin-echo 3D-SPACE (three-dimensional sampling perfection with application-optimized contrasts using different flip angle evolutions) sequence delineated the total inner ear fluid space from the surrounding bone according to the method suggested by Ref. [2].

The T2-weighted, 3D-FLAIR was characterized by the following parameters: TR 6000 ms; TE 134 ms; TI 2240 ms; FA 180°; FOV 160 × 160 mm^2^; 36 slices; base resolution 320; averages 1; slice thickness 0.5 mm. The high-resolution, strongly T2-weighted, spin-echo 3D-SPACE sequence of the temporal bone had the following parameters: TR 1000 ms; TE 133 ms; FA 100°; FOV 192 × 192 mm^2^; 56 slices; base resolution 384; averages 4; slice thickness of 0.5.

#### Semi-quantitative (visual) grading of the endolymphatic space

ELH was observed as enlarged negative-signal spaces inside the labyrinth and cochlea on the 3D-FLAIR images [2]. Semi-quantitative (SQ) visual grading of the ELS (*sq*ELS) was performed independently by an experienced head and neck radiologist (SSt) and a neurologist (JG, VK) who were blinded to the clinical patient data. The ELS’s characterization in the vestibulum and cochlea was based on a previously described 4-point [7, 32] ordinal scale classification.

#### 3D-(volumetric) quantification of the endolymphatic space.

3D- or volumetric quantification of the ELS (*v*ELS) was achieved in two steps: First, segmentation of the total fluid space (TFS) was based on IE-Vnet [33], a recently proposed and pre-trained volumetric deep learning algorithm with V-net architecture. IE-Vnet was deployed via the TOMAAT module [34] into the 3D–Slicer toolbox (version 4.11, [35]).

Second, ELS and perilymphatic space (PLS) were differentiated within the TFS using volumetric local thresholding (VOLT, [36]) that uses ImageJ Fiji [37], including the “Fuzzy and artificial neural networks image processing toolbox” [38] and the “MorphoLibJ Toolbox” [39]. The resulting 3D volume included ELS and PLS classifications for cochlea and vestibulum (cutoff 6). The ELS ratio, $ER [\%]=\frac{ELS}{TFS}\times 100$, was calculated analogously to Ref. [40]. ELS symmetry between both inner ears was assessed using the absolute value of the asymmetry index *AI* [%] = $\frac{({ELS}_{right}-{ELS}_{left})}{({ELS}_{right}+{ELS}_{left})}\times 100$.

### Statistics and validation parameters

Statistical analyses were performed using the Statistical Package for Social Sciences software (SPSS, Inc, Chicago, IL, USA). Categorical values are reported as the number of cases that fit the category/number of participants with normal vestibulocochlear testing [%]; ordinal or scalar values are presented as (mean ± standard deviation). Results were reported at a significance level of *p* < 0.05 and *p* < 0.01. In addition, the linear agreement between parameter pairs was calculated using two-sided Pearson’s correlation coefficient.

## Results

A detailed clinical and neurophysiological characterization of the group of participants with normal vestibulocochlear testing (^*vc*^HP) is provided in Table 1. No participant needed to be excluded due to vestibulocochlear dysfunction.

**Table 1:** Clinical and neuro-otological characterization of vestibulocochlear healthy participants

	^*vc*^HP
Participants	*n* = 32
Age [in years]	45.8 ± 17.2
Age range [years]	21–75
Gender	20 females
Handedness	30 RH, 2 LH
TN	0/32
SPN	0/32
OT	0/32
SVV deviation	0/32
HIT pathological	0/31 (0%)
HIT mean gain	1.0 ± 0.05 (0.9–1.1)
HIT AI [%]	4.0 ± 2.7 (0–11.1)
CS pathological	0/32 (0%)
CS mean [°/s]	17.8 ± 6.7 (8.2–33.8)
CS AI [%]	10.6 ± 7.1 (1.2–25.5)
PTA pathological	2/32 (6.3%)
Presbycusis	2/32 (6.3%)
PTA mean [dB]	24.8 ± 10.1

AI =  asymmetry index; CS =  caloric stimulation; HIT =  head impulse test; ^vc^HP =  participants with normal vestibulocochlear testing; LH =  left-handed; OT =  ocular torsion; PTA =  pure tone audiometry; RH =  right-handed; SPN =  spontaneous nystagmus; SVV =  subjective visual vertical; TN =  triggered nystagmus (by head shaking, and/or hyperventilation)

Following a 4-point ordinal scale [7, 32], a mild ELH (grade 1) was found in 21/64 (32.8%) ears uni- or bilaterally in either cochlear, vestibulum, or both ($sq{ELS}_{cochlea}^{HC}$: grade 0.2 $\pm$ 0.4, $sq{ELS}_{vestibulum}^{HC}$: grade 0.3 $\pm$ 0.5, $sq{ELS}_{inner ear}^{HC}$: grade 0.2 $\pm$ 0.3, range: 0–1). This means that the extent of ELH was low ($v{ELS}_{cochlea}^{HC}$: 2.8 $\pm$ 1.1 mm^3^, $v{ELS}_{vestibulum}^{HC}$: 6.6 $\pm$ 2.1 mm^3^, $v{ELS}_{inner ear}^{HC}$: 9.4 $\pm$ 2.3 mm^3^). A detailed description of the ELS quantification results can be viewed in Table 2.

**Table 2:** Semi- and 3D-quantification of the endolymphatic space

	^*vc*^HP
Ears	*n* = 64
Cochlea	
ELH	10/64 (15.6%)
Side	
Unilateral	4/32 (12.5%)
Bilateral	3/32 (9.4%)
* sq*ELS [grade]	0.2 ± 0.4 (0–1)
* v*ELS [mm^3^]	
Mean	2.8 ± 1.1 (0.3–6.6)
AI	20.2 ± 12.9 (1.0–54.4)
ER	3.1 ± 1.0 (0.6–5.1)
TFS [mm^3^]	89.5 ± 12.0 (59.4–126.9)
Vestibulum	
ELH	16/64 (25%)
Side	
Unilateral	4/32 (12.5%)
Bilateral	6/32 (18.8%)
* sq*ELS [grade]	0.3 ± 0.5 (0–2)
* v*ELS [mm^3^]	
Mean	6.6 ± 2.1 (2.4–11.7)
AI	15.2 ± 10.5 (1.0–33.1)
ER	3.7 ± 0.8 (1.9–5.4)
TFS [mm^3^]	180.7 ± 21.2 (140.4–233.9)
Inner ear	
ELH	21/64 (32.8%)
Side	
Unilateral	4/32 (12.5%)
Bilateral	9/32 (28.1%)
* sq*ELS [grade]	0.2 ± 0.3 (0–1)
* v*ELS [mm^3^]	
Mean	9.4 ± 2.3 (4.5–13.4)
AI	12.7 ± 7.6 (1.6–25.4)
ER	3.5 ± 0.6 (2.4–4.7)
TFS [mm^3^]	270.2 ± 29.0 (216.3–329.9)

AI  =  asymmetry index; ELH =  endolymphatic hydrops; ELS =  endolymphatic space; ER =  endolymphatic ratio  =  ELS/TLS [%]; sqELS =  semi-quantitative or visual quantification of the ELS following a 4-point ordinal scale classification [7]; vELS =  volumetric or 3D quantification of the ELS [mm^3^]; ^vc^HP =  participants with normal vestibulocochlear testing; TFS =  total fluid space

**Fig. 1:**
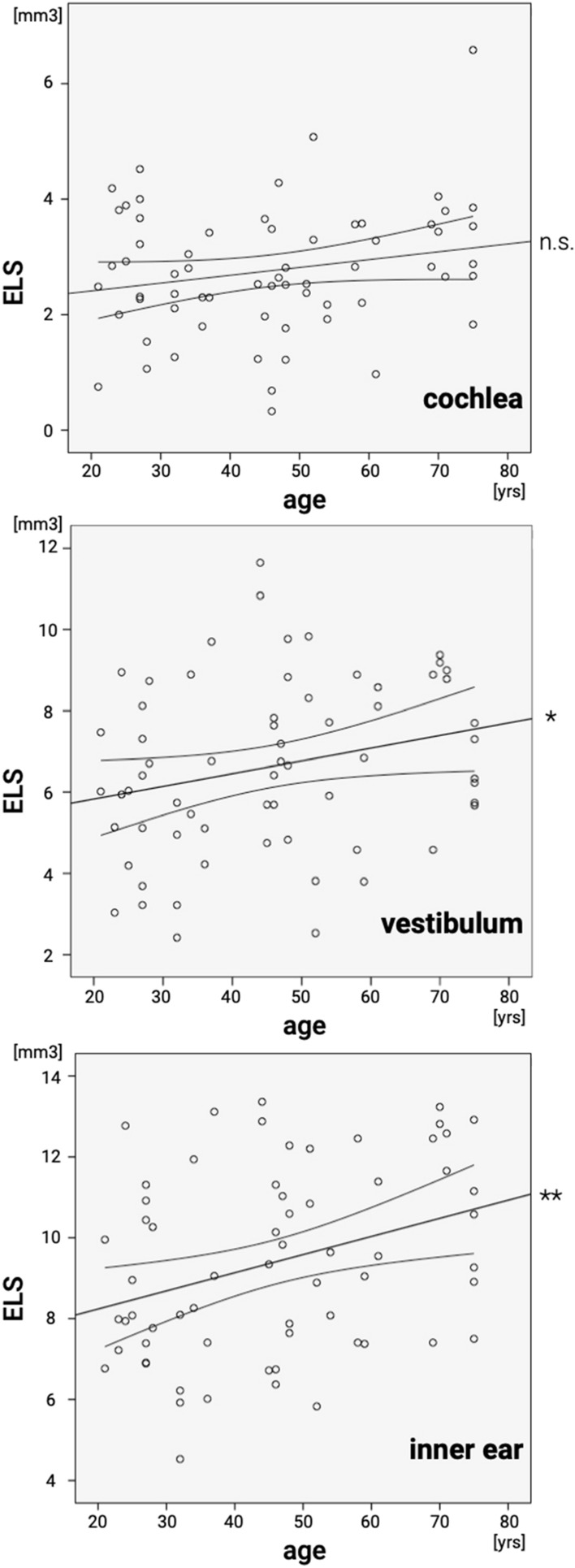
Scatter plot for age and 3D quantification of the endolymphatic space. Correlation results for cochlea (top), vestibulum (middle) and inner ear (bottom) of participants with normal vestibulocochlear testing: age and 3D-(volumetric)-quantification (vELS) of the endolymphatic space (ELS) were found to be moderately strong positively correlated for the cochlea, *r*(64) = 0.21, *p* = 0.100 (linear slope = 0.043), vestibulum, *r*(64) = 0.25, *p* = 0.045 (linear slope = 0.063), and inner ear, *r*(64) = 0.33, *p* = 0.008 (linear slope = 0.109). Significant linear agreements are marked (*) for a significance level *p* < 0.05 and (**) for a significance level *p* < 0.01

Age and *v*ELS were found to be significantly positively correlated for the inner ear, *r*(64) = 0.33, *p* = 0.008, and vestibulum, *r*(64) = 0.25, *p* = 0.045. For the cochlea, values correlated positively without reaching significance, *r*(64) = 0.21, *p* = 0.100. The corresponding scatter plots are depicted in Fig. 1.

## Discussion

The current study investigated age-dependent changes in the endolymphatic space (ELS) in 32 participants with normal vestibulocochlear testing (Table 2). Mild ELH (grade 1) was found in 15.6% in the cochlea (thereof 12.5% unilateral and 9.4% bilateral), 25% in the vestibulum (thereof 12.5% unilateral and 18.8% bilateral), and 32.8% when considering the inner ear (thereof 12.5% unilateral and 28.1% bilateral). None of the participants’ semi-quantitative ELH classification grades (*sq*ELS) exceeded grade 1 (mild ELH) following a 3-point [41] and 4-point [7] ordinal scale. Mean ELS volume (*v*ELS) was 2.8 µl for the cochlea, 6.6 µl for the vestibulum, and 9.4 µl for the inner ear. ELS ratio (ER = $\frac{ELS}{TFS}\times 100$) remained below 5% in cochlea (3.1%), vestibulum (3.7%), and inner ear (3.5%). Asymmetry index remained beneath 25% in cochlea (20.2%), vestibulum (15.2%), and inner ear (12.7%). ELS volume showed age-dependent significant changes for the inner ear, *r*(64) = 0.33, *p* = 0.008, and vestibulum, *r*(64) = 0.25, *p* = 0.045 (cp. Fig. 1). For the cochlea, values correlated positively without reaching significance, *r*(64) = 0.21, *p* = 0.100.

In the following, the mentioned results will be discussed given previous studies conducted on ELS in “healthy” participants.

### ELH prevalence in “healthy” participants

The results of the current study fit well with the findings in the literature. An overview of ELH prevalence in studies on the “healthy” population to date is shown in Table 3. Notably, even excluding studies using the non-affected ear in unilateral Meniere's disease (*u*MD) as a healthy control group, a relatively high ELH prevalence was found. In *i*MRI studies (see Table 3b) using semi-quantitative three-point visual grading [41], *mild* ELH was reported to lie between 3.3 and 28.6% of participants in the cochlea [10, 42], 6.7–25% in the vestibulum [7, 43], and 7.5–10% in the inner ear [7, 43]. *Significant* ELH was reported between 9.5 and 13.3% in the cochlea [10] and up to 30% in the vestibulum [42]. In contrast, in *h*TB studies' adult population (see Table 3a), including participants with MD, ELH prevalence was found to be around 9% [44], and without MD, around 4.5% [6].

**Table 3:** Literature overview of ELH prevalence in "healthy" participants

Study	Year [reference]	Kind of subjects	Nr. Of subjects	Age [years]	Vestibulocochlear testing	Method of quantification	ELH		
							Cochlea	Vestibulum	Inner ear
(a) Human temporal bone studies									
Bachor and Karmody	1995 [57]	I	70	0–10	No	sq_h_, V	16.9%	–	–
Belal and Atunel	1980 [44]	A^+^	357	0–98	No	sq_h_, V	9%	–	–
Buch	1966 [56]	N	73	< 0	No	sq_h_, V	0.7%	–	–
Merchant	2005 [6]	A^∆^	963	Cases: 6–91, rest: n.s	No	sq_h_, V	4.5%	–	–
(b) iMRI, *intravenous*									
Attyé	2017 [42]	HP	30	58.7 ± 10.9 (range: > 40)	No	sq_3_, V	13.3%, ^■^	30%, ^■^	n.s.
Boegle	2021 [7]	^*vc*^HP	33	46.4 ± 15.6 (range: 21–75)	*Neuro-orthoptic assessment, SVV, VOG during CS & HIT, c/oVEMPs, PTA*	sq_3+4_, V	9%, ^☐^	25%, ^☐^	7.5%, ^☐^
Ito	2016 [43]	HP	15	57.7 ± 17.9 (range: 20–76)	No	sq_3_, V	3.3%	6.7%	10%
Yoshida	2018 [10]	^*c*^HP	21	56.1 ± n.s. (range: 24–79)	PTA	sq_3_, M	9.5^■^–28.6^☐^ %	0^■^–7.1^☐^ %	n.s.

This table has no claim to be exhaustive. Inclusion criteria for this table were the investigation of “healthy” controls and the usage of an established, comparable method for the semi-quantitative classification of the endolymphatic hydrops (*sq*ELH). For the *h*TB studies, the 3-point *sq*ELH grading system of Schuhknecht et al. [45] was used. For the *i*MRI studies, the 3-point *sq*ELH grading system of Nakashima et al. [41] was used. Here, “healthy” control includes patients without audiological or vestibular problems and excludes studies using the non-affected ear in unilateral Meniere's disease as a healthy control group

+  = including patients with Morbus Meniere, MD; ■ = significant ELH following sp_3_ [41]; ☐ = mild ELH following sp_3_ and sp_4_; ∆ = using the human temporal bone collection at the Massachusetts Eye and Ear Infirmary; A = adult population including normal ears and cases with otological diseases (for more detail see [6]); ^c^HP = participants with nonotological diseases (for more detail, see [10]) without audiological, vestibular or neurological problems; ^vc^HP = participants with neurological diseases (for more detail, see [7]) with normal vestibulocochlear testing; c/oVEMP = ocular/cervical vestibular evoked myogenic potential; CS = caloric stimulation; HP = healthy participants with no history of audiological, vestibular, or neurological problems (for more details, see [43]); HIT = head-impulse test; I = infant population including congenital anomalies (51.4%), infectious diseases (17.1%), non-infectious diseases (18.6%), and tumors (12.9%); N = newborn mainly premature population; n. s. = not specified; PTA = pure tone audiometry; sq_3_ = semi-quantitative following 3-point classification in cochlea and vestibulum following [41]; sq_4_ = semi-quantitative following 4-point classification in cochlea and vestibulum following [7], sq_h_ = semi-quantitative following 3-point histological classification of the cochlea following [45]; SVV = subjective visual vertical; TFS = total fluid space; V = visual assessment

In summary, the overlap of the 3-point *sq*ELH grading system of Schuhknecht et al. [45] for the *h*TB studies and of Nakashima et al. [41] for the *i*MRI helps to compare their results, a direct transfer of the results should be done with caution. After all, while *i*MRI can only indirectly verify ELH, it provides in-vivo longitudinal ELS visualization with multi-slice 3D-quantification in addition to contemporary measurement of vestibulocochlear function. *h*TB studies, on the other hand, provide direct identification of the distension of Reissner's membrane and further histopathologic evidence for MD.

### ELS volume in “healthy” participants

The current study's ELS volume was low in the cochlea 2.8 $\pm$ 1.1 mm^3^, vestibulum 6.6 $\pm$ 2.1 mm^3^, and inner ear 9.4 $\pm$ 2.3 mm^3^ compared to volumes in the previous literature, especially across methods in both *h*TB [23, 46, 47] and high-resolution CT [48–50]. A detailed description of the ELS 2D-(area) and 3D-(volumetric) quantification in previous *h*TB and *i*MRI studies on the “healthy” population to date can be viewed in Table 4.

**Table 4:** Literature overview of previous ELS 2D- and 3D-quantification results in “healthy” participants

Study	Year [reference]	Kind of subjects	Nr. of subjects	Age [years]	Vestibulo- cochlear testing	Method of visualization, quantification	Cochlea		Vestibulum		Inner ear	
							ELS	TFS	ELS	TFS	ELS	TFS
(a) 2D- (area) quantification of the ELS [mm^2^] and area ratio (AR) in [%]												
Boegle	2021 [7]	^*vc*^HP	33	46.4 ± 15.6	*see below*	*iv*, A	6.7 ± 3.5%		11.9 ± 4.4%		9.6 ± 2.9%	
							1.1 ± 0.6	16.0 ± 1.7	2.5 ± 1.0	20.5 ± 2.4	3.5 ± 1.3	31.4 ± 3.5
Cho	2021 [54]	HP^∆^	10	n.s	PTA	*hTB,* M	6.4 ± 2.2%		28.9 ± 6.2%		n.s.	
Liu	2011 [51]	HP	20	23.5 ± 1.9	*PTA, tympanometry, EcochG, VEMP*	*it,* M	9–28%		14–40%		n.s.	
	2012 [52]	HP	20	48.9 ± 2.95		*it,* M	8–26%		20–41%		n.s.	
	2015 [53]	HP	20	37.2 ± 11.0		*it,* M	7–27%		17–39%		n.s.	
(b) 3D- (volumetric) quantification of the ELS in [µl, mm^3^] and ELS ratio (ER or ELS/TFS) in [%]												
Boegle	2021 [7]	^*vc*^HP	33	46.4 ± 15.6	*neuro-orthoptic assessment,* *SVV, VOG during CS and HIT, o/cVEMPs, PTA*	*iv,* A	5.5 ± 2.1%		6.2 ± 1.5%		6.0 ± 1.4%	
							5 ± 2.2	90.0 ± 13,5	11.1 ± 3.3	178.65 ± 22,5	16.2 ± 5.8	268.7 ± 31.5
Inui	2016 [58]	CRS	24	57.5 ± n.s	no	*iv,* M	8.8 ± 5.3%		16.2 ± 9.0%		11.7 ± 5.7%	
							10.3 ± 6.9	114.9 ± 13.6	11.5 ± 7.1	69.8 ± 10.6	32.6 ± 16.6	279.8 ± 33.5
	2021 [40]	HP	100	57.8 ± n.s	no	*iv,* M	10.3 ± 6.7%		17.3 ± 12.2%		13.7 ± 7.8%	
							11.8 ± 8.3	112.9 ± 15.9	12.2 ± 7.5	69.1 ± 9.9	39.2 ± 22.0	282.1 ± 33.2
Ito	2019 [55]	HP	47	58.4 ± 16.3	no	*iv,* M	10.2 ± 6.8%		17.7 ± 10.2%		13.9 ± 7.9%	
							–	–	–	–	–	
Teranishi	2009 [46]	^*c*^HP^∆^	5	66.2 ± 6.3	no	*hTB,* A	10.8 ± 3.6%		24.1 ± 10.7%		19.8 ± 13.0%	
							5.1 ± 0.6	47 ± 6.6	24.4 ± 3.6	99.8 ± 17.9	29.1 ± 4.1	146.8 ± 21.9

This table has no claim to be exhaustive. Inclusion criteria were the investigation of "healthy" participants and the usage of an established, comparable method for a two-dimensional (2D) or three-dimensional (3D) quantification of the endolymphatic space (ELS). For 2D-quantification (or areal quantification) results are presented in [mm^2^], and normalized as area ratio (AR or 2D ELS/TFS) in [%]. For 3D-quantification (or volumetric quantification) results are presented in [µl, mm^3^] and normalized as ELS ratio (ER or 3D ELS/TFS) in [%]. In this table, “healthy” participants include patients without audiological or vestibular problems and exclude studies using the non-affected ear in unilateral Meniere's disease as a “healthy” participant group

∆ = using the human temporal bone collection at the Massachusetts Eye and Ear Infirmary; A = automatic measurement; AR = area ratio or 2D ELS/TFS) in [%]; ^c^HP = participants with nonotological diseases (for more detail, see [46]) and without audiological, vestibular or neurological problems; ^vc^HP = participants with neurological diseases (for more detail, see [7]) with normal vestibulocochlear testing; CRS = chronic rhinosinusitis; CS = caloric stimulation; EcochG = electrocochleography, ELS = endolymphatic space; ER = ELS ratio or 3D ELS/TFS in [%]; HP = healthy participants; it = intratympanic iMRI; iv = intravenous iMRI; HIT = head-impulse test; hTB = human temporal bone study; M = manual measurement using software; MRI = magnetic resonance imaging; n. s. = not specified; o/cVEMP = ocular/cervical vestibular evoked myogenic potential; SVV = subjective visual vertical; TFS = total fluid space; V = visual assessment

It is noticeable that the lower volumetric values or ratios occur in the studies with participants that were controlled for vestibulocochlear testing. In addition, the absolute values of both ELS quantification methods (although 2D- more than the 3D- ELS quantification method) are variable across the different studies, and relative size ELS are less variable (with ELS-Ratio being most constant, also across methods).

### ELH laterality and ELS symmetry

In healthy participants, cochlear and vestibular ELH laterality (bi- or unilateral) and ELS symmetry (when using 2D or 3D quantification) were omitted. Investigated *h*TB and *i*MRI studies either only have one ear at their disposal [46, 51–53], limit information on laterality to the patient cohort [6, 10, 42–44, 54, 55], or do not discuss ELH laterality as a category [40, 56–58]. The current study showed 12.5% unilateral mild ELH in the cochlea and vestibulum, 9.4% showed a mild bilateral ELH in the cochlea, and 18.8% a mild bilateral ELH in the vestibulum, while the asymmetry index remained < 25% (cochlea: 20.2%, vestibulum 15.2%, inner ear: 12.7%). Laterality and symmetry add to the characteristic pattern in “healthy” participants, alongside volumetric extent and maximally mild ELH in semi-quantitative ELS quantification.

Therefore, ELS studies should include semi-quantitative (SQ) grading and 3D quantification. SQ grading should include the 3-point grading scale by Nakashima et al. [41] as it is the most commonly used grading system and can serve as a calibration point. Reported 3D-quantification values should include symmetry parameters, such as asymmetry indices for un-normalized data and the relative size of the ELS for normalized data.

### ELS age-dependency

Age and *v*ELS were found to be significantly positively correlated for the inner ear (*r*(64) = 0.33, *p* = 0.008), and vestibulum (*r*(64) = 0.25, *p* = 0.045). For the cochlea, values correlated positively without reaching significance *(r*(64) = 0.21, *p* = 0.100)(Fig. 1). These results seem plausible because of the increased vestibulocochlear dysfunction with age [59–62], including the frequent cases without the patient noticing the vestibular dysfunction occurred [63–65]. Contrary, two published studies by Inui et al. [40, 58] showed that participants < 60 years had significantly larger TFS and ELS/TFS volume ratios in the inner ear and significantly larger ELS/TFS volume ratios in the vestibulum in comparison to the participant group ≥ 60 years. The differing results between the current study and the latter might be explained by the difference in methods or the selection of participants (Table 4b).

The overall mild degree in ELS increase without symptoms and (in general) normal audiovestibular testing results also further question the current definition of ELH. The word hydrops implies an excessive pathologic accumulation of endolymphatic fluid within the ELS. However, the current results imply that a mild ELH might not necessarily be a pathologic sign but can represent a physiological norm, especially with increasing age and when unaccompanied by audiovestibular symptoms or (diagnostic) signs of audiovestibular dysfunction. An objective description of different ELS distention grades without statement on its pathogenicity (± ELH) would be preferable. An easy solution would be to simply retain all previous suggestions of semi-quantitative ELH grading as ELS grading, and discard the statement referring to no, mild, moderate or severe ELH.

### Methodological limitations of the study

Two major limitations of the current study need to be considered: first, the limited number of subjects included, and second, the circumstance that *i*MRI imaging could only be done in patients with other (neurological) pathologies. A representative ELS study with a higher number of completely healthy subjects, ideally of 20 participants per decade, ranging between 20 and 90 years of age, would be desirable and is still pending. Second, influences of the participants' underlying neurological pathologies on the ELS appear unlikely (view inclusion and exclusion criteria, Sect. 2.2.) but cannot entirely be excluded. In addition, two of our participants showed signs of presbyacusis in our audiometric testing, and we do not know if hearing performance influences the ELS. However, ethical considerations did not allow us to include healthy volunteers without a medical indication for an iMRI with contrast agent. The decision to avoid unnecessary contrast agent application was based on prior findings of signal intensity increases in the dentate nucleus and globus pallidus on T1-weighted MR images after applying MR contrast agents that are still under investigation [66–68]. Third, the study lacks histological confirmation of endolymphatic hydrops since the in-vivo acquisition of histological specimens is currently not possible.

## Conclusion

Age-dependent increases of the ELS should be considered when evaluating potential ELH in single subjects and statistical group comparisons.

## Abbreviations

3D: Three-dimensional
AI: Asymmetry index
ELH: Endolymphatic hydrops
ELS: Endolymphatic space
FLAIR: Fluid-attenuated inversion recovery
HIT: Head-impulse test
^*vc*^HP: Participants with normal vestibulocochlear testing
*h*TB: Human temporal bone
IE-Vnet: Inner ear Vnet
iMRI: Delayed intravenous gadolinium-enhanced MRI of the inner ear
MD: Ménière’s disease
MRI: Magnetic resonance imaging
PLS: Perilymphatic space
PTA: Pure tone audiometry
SPN: Spontaneous nystagmus
SVV: Subjective visual vertical
TFS: Total fluid space
*u*MD: Unilateral MD
VOG: Video-oculography
